# Metformin Prevents Follicular Atresia in Aging Laying Chickens through Activation of PI3K/AKT and Calcium Signaling Pathways

**DOI:** 10.1155/2020/3648040

**Published:** 2020-11-26

**Authors:** Jinwei Yao, Yanfen Ma, Shuo Zhou, Tingting Bao, Yuling Mi, Weidong Zeng, Jian Li, Caiqiao Zhang

**Affiliations:** College of Animal Sciences, Zhejiang University, Hangzhou 310058, China

## Abstract

Increased follicular atresia occurs with aging and results in reduced fecundity in laying chickens. Therefore, relieving follicular atresia of aging poultry is a crucial measure to maintain sustained high laying performance. As an antiaging agent, metformin was reported to play important roles in preventing aging in diverse animals. In this study, the physiological state of the prehierarchical follicles in the peak-laying hens (D280) and aged hens (D580) was compared, followed with exploration for the possible capacity of metformin in delaying atresia of the prehierarchical follicles in the aged D580 hens. Results showed that the capacity of yolk deposition within follicles declined with aging, and the point of endoplasmic reticulum- (ER-) mitochondrion contact decreased in the ultrastructure of the follicular cells. Meanwhile, the expression of apoptosis signaling genes was increased in the atretic small white follicles. Subsequently, the H_2_O_2_-induced follicular atresia model was established to evaluate the enhancing capacity of metformin on yolk deposition and inhibition of apoptosis in the atretic small white follicles. Metformin inhibited apoptosis through regulating cooperation of the mitochondrion-associated ER membranes and the insulin (PI3K/AKT) signaling pathway. Furthermore, metformin regulated calcium ion homeostasis to relieve ER-stress and inhibited release of mitochondrion apoptosis factors (BAD and caspase). Additionally, metformin activated PI3K/AKT that suppressed activation of BAD (downstream of the insulin signaling pathway) in the atretic follicles. Further, serum estrogen level and liver estrogen receptor-*α* expression were increased after dietary metformin supplementation in D580 hens. These results indicated that administration of dietary metformin activated the PI3K/AKT and calcium signaling pathway and enhanced yolk deposition to prevent chicken follicular atresia.

## 1. Introduction

Laying hens exhibit a rapid reduction of egg-laying rate as they start aging. At 80 weeks of age, hens experience a significant decrease in egg production, and their ovarian functions markedly decline [1, 2]. The decreasing egg production in D580 hens is predominantly attributed to three reasons: (1) accelerated loss of prehierarchical follicles (PHFs) in the aged ovaries, (2) increased atresia experienced by PHFs; (3) reduced capacity of yolk precursor formation in the liver and decreased yolk deposition within the PHFs [3, 4]. A follicle is mainly comprised of three types of cells: one oocyte and two kinds of somatic cells (GCs for the granulosa cells and TCs for the theca cells). GCs play a fundamental role in follicular atresia. In the atretic follicles (AFs), it is observed that apoptosis occurs earlier in GCs than in oocytes and TCs [5, 6]. Based on a previous study, there is a more significant decrease in the number of PHFs in D580 hens than in D280 hens, and the decreasing number of follicles undergo atresia [7]. Therefore, it is important to prevent or postpone follicular atresia to maintain competitive laying performance.

The decision of apoptosis or survival of GCs is reported to be closely related with multiple signaling pathways. Inhibition of the PI3K/AKT in the insulin signaling pathway would accelerate apoptosis of GCs and lead to premature ovarian failure [8, 9]. On the contrary, activation of PI3K stimulated differentiation of GCs in follicles [10]. In terms of promoting the survival of GCs, AKT was believed to assist follicle-stimulating hormone (FSH) to stimulate the secretion of steroid hormones and the proliferation of the GCs [11, 12]. However, little is known about the functions of the PI3K/AKT signaling pathway in the development and growth of chicken ovarian follicles, especially its functions in preventing follicular atresia, which is crucial for improving egg reproduction.

Activated endoplasmic reticulum stress (ER-stress) and the mitochondrion apoptosis pathway are deleterious for GC survival [13]. Firstly, overactivation of ER-stress and activation of the mitochondrion apoptosis signaling pathway could trigger an intracellular calcium ion imbalance [14]. It is well known that calcium transduction plays a pivotal role in controlling the fate of senescent cells [15]. Mitochondrion-associated ER membranes (MAMs) are the functional domains in these two organelles that are involved in Ca^2+^ exchange [16]. MAM is composed of glucose regulatory protein 75 (GRP75) and the inositol 1,4,5 trisphosphate (IP_3_) receptor channel (IP_3_R, the channel of Ca^2+^ release from the ER) that is associated with the mitochondrion calcium uniporter (MCU, IP3R-GRP75-MCU calcium signaling channel) [17]. The transportation of calcium from MAM is critical for regulating mitochondrion function and energy metabolism [18]. Knockdown of GRP75 reduced the ability of ER to carry Ca^2+^ to the mitochondrion [19]. Secondly, the unphosphorylated BAD (ATK downstream transcription factor) induced the release of cytochrome C (Cyt *c*) and ultimately activated caspase, leading to apoptosis of the mitochondrion. This function is accomplished by increasing the mitochondrion calcium level (calcium overload) [20, 21]. However, just what the involvement of the calcium transportation pathway and MAM is in the apoptosis of aging-induced follicular atresia is still unclear.

The yolk formation and deposition depend mainly on the liver-blood-ovarian axis under the regulation of multiple hormones, but predominantly by estrogen (E_2_) that stimulates apolipoprotein B (ApoB) and vitellogenin II (VTGII) synthesis in the liver through binding with estrogen receptor *α* (ER-*α*) [22, 23]. Recent studies demonstrate that the decline of the serum E_2_ level during the chicken aging process eventually decreases the ability of yolk formation [24]. In yolk deposition, the main components of the yolk precursors are very-low-density lipoprotein (VLDL) and vitellogenin (VTG). VLDL is synthesized in the liver and subsequently transported into the oocytes through VLDLR-mediated endocytosis [25]. It had been reported that VLDLR existing on surface of migrating neuroblasts could activate PI3K/AKT through p-Dab1 [26]. Furthermore, the peroxisome proliferator-activated receptor (PPAR) is an important factor for regulating adipocyte differentiation and function, and it plays a crucial role in glycerol production as well as lipid uptake, synthesis, storage, and hydrolysis [27]. Increased expression of PPAR-*γ* enhanced the ability of lipid transport and uptake in the corresponding cells of laying hens [25]. In addition, the occludin protein, which is located in the periphery of GCs, would inhibit the transport of yolk precursors by sealing gap junctions between GCs [28, 29]. Although it was shown that aging in laying hens was accompanied by a decline in yolk synthesis [24], the declined ability of follicular yolk deposition in aging hens needs to be further assessed, especially in the follicles that are initiating atresia.

Metformin (Met), a derivative from an herbal medicine named *Golega officinalis*, is used for treating lactone of livestock in animal husbandry [30]. Recently, Met has been demonstrated to play an important role in alleviating aging [31]. On one hand, Met was reported to postpone the ovarian aging process in mice by inhibiting follicular loss and maintaining follicular reserve. In addition, it could relieve ovarian oxidative stress as well as production of mitochondrion ROS [32, 33]. On the other hand, Met was able to decrease the expression of GRP78 (a kind of ER-stress marker) which was induced by the aging-inducing drug D-galactose [33]; therefore, Met could prevent apoptosis that was induced by ER-stress. However, it is unknown whether Met can cause apoptosis to decline by maintaining the balance of Ca^2+^ concentration/participating in MAM, although it has been reported to relieve ER-stress/mitochondrion-stress [34]. Additionally, Met relieved the polycystic ovary syndrome by stimulating the PI3K/AKT pathway [35]. Meantime, Met alleviated vascular smooth muscle inflammation which is provoked by lipopolysaccharide via promoting the expression of PPAR-*γ* [36]. However, the function of Met in regulating yolk deposition of the AFs is unknown. Furthermore, it is unclear whether Met can reduce follicular atresia through the PI3K/AKT pathway.

In this study, the changes in yolk deposition and expression of apoptosis-associated genes or proteins are compared among D280-SWFs (small white follicles, 2-4 mm, a kind of PHF), D580-SWFs, and atretic small white follicles (ASWFs) from D580 hens. In order to reveal the mechanism of follicular atresia and the role of Met in retarding follicular atresia (including yolk deposition and cell apoptosis), a model of ASWFs is established by induction with H_2_O_2_. A PI3K inhibitor (Taselisib) is used to inhibit PI3K in ASWFs to explore the role of Met in the PI3K/AKT pathway. The results may provide a foundation for preventing ovarian aging to prolong the laying period in laying poultry.

## 2. Materials and Methods

### 2.1. Animals

Hyline white hens (D280 and D580) were obtained from a local chicken farm and were maintained in cages with free access to feed and water, under a controlled photoperiod of 14 h light : 10 h dark cycles. All experimental procedures were conducted following the Guiding Principles for the Care and Use of Laboratory Animals of Zhejiang University (ZJU20170660).

### 2.2. Collection and Culture of PHFs

Ovaries were separated from D280 and D580 hens for collection of PHFs. The follicles were washed three times with ice-cold phosphate-buffered saline (PBS) for the following experiments. Single PHFs were cultured in 24-well culture plates containing 500 *μ*L complete Dulbecco's modified Eagle's medium (DMEM high glucose, SH30243.01) with 5% fetal calf serum (FCS; HyClone, Tauranga, New Zealand), 10 *μ*g/mL insulin, 5 *μ*g/mL transferrin, 30 nM selenite (ITS, Sigma-Aldrich, MO, USA), 100 IU/mL penicillin, and 100 *μ*g/mL streptomycin at 38.5°C and 5% CO_2_ for 72 h. The media were renewed every 24 h.

### 2.3. Treatments with Chemicals

For the in vitro experiment, there were three treatments for the PHFs: (a) To establish an ASWF model, H_2_O_2_ was added into the medium at different concentrations (0, 0.1, 1, and 10 mM; Sinopharm Chemical Reagent Co. Ltd., 10011218, Shanghai, China). (b) For screening the optimal concentration of Met (in the form of metformin hydrochloride, M107827, Aladdin), the H_2_O_2_-induced ASWFs were treated with different concentrations of Met (0, 0.2, 2, and 20 mM). (c) For detection of the function of Met in the insulin signaling pathway (PI3K/AKT), the atretic follicles were treated with 2 mM Met and 0.3 mM Taselisib (a PI3K inhibitor; synonyms: GDC-0032, RG-7604, and MCE) alone or in combination. For the incorporation of bromodeoxyuridine (BrdU, 19-160, Sigma-Aldrich, WI, USA), PHFs were incubated for 24 h with BrdU for 72 h, then PHFs were collected for subsequent determinations. For the in vivo experiment, thirty D580 hens (weight ~ 2 kg) were chosen randomly and divided evenly into two groups (experimental/control group). Each hen from the experimental group was given a sustained-release Met tablet (H31021359, Shanghai Hengshan Pharmaceutical Co. Ltd.) at 62.5 mg/kg (BW) with 3 mL pure water every day for seven consecutive days (according to daily laying rate, Supplemental Figure 1B). The control group was given an equal volume of pure water. Blood samples were collected from the wing veins for the preparation of plasma and serum on the 8th day. The hens were then sacrificed by cervical bleeding after anesthesia with pentobarbital sodium, and tissue samples (livers and PHFs) were taken for subsequent experiments.

### 2.4. Morphological Observation

Follicles were fixed in 4% paraformaldehyde for 24 h at 4°C and dehydrated with graded ethanol, subsequently embedded in paraffin at 60°C, and sectioned with a thickness of 4 *μ*m. H&E staining was performed according to a standard histological procedure [13]. Immunofluorescence or immunohistochemical staining (IF/IHC) was performed as previously reported [37]. The primary antibodies in IF/IHC are as follows: rabbit anti-caspase3 (1 : 100, ET1602-39), anti-cytochrome C (1 : 100, ET1610-60), anti-mitochondrion fusin 2 (1 : 100, ER 1802-23), anti-PI3K (1 : 100, Et1608-70), mouse anti-BAD (1 : 50, RT1067), anti-GRP75 (1 : 50, M1603-1, HuaBio, Hangzhou, China), rabbit anti-VLDLR (1 : 100, MAB2258), mouse anti-ER-*α* (1 : 200, NB300-560, Novus Biologicals, USA), anti-occludin (1 : 50, sc-133256, Santa Cruz Biotechnology Inc., Santa Cruz, USA), and anti-BrdU antibody (1 : 200, AB_2314035, G3G4; DSHB, Iowa (IA), USA). The fluorescence images of the slides were visualized using a fluorescence microscope (Olympus IX70, Tokyo, Japan). The results of IHC and H&E staining were observed under a microscope (Eclipse 80i, Nikon, Japan), and the images were photographed with a digital camera (DS-Fi1, Nikon, Japan).

### 2.5. TUNEL Assay

TUNEL assay was performed using a TUNEL assay kit (Vazyme Biotech Co. Ltd., Nanjing, China) following the manufacturer's protocol. TUNEL-positive cells were marked as green.

### 2.6. Measurement of Blood Biochemical Parameters

Serum E_2_ was determined using an E_2_ ELISA test kit (ERK R7005, Endocrine Technologies, Inc., San Francisco, CA, USA) according to the manufacturer's instructions. The levels of plasma triacylglycerol (TG, A111-1-1, in the range of 0-10.34 mM, *r*^2^ > 0.995), high-density lipoprotein (HDL, A112-1-1, in the range of 0.065-3.8 mM, *r*^2^ > 0.995), free fatty acids (FFA, A042-2-1, in the range of 9.1-2000 *μ*M, *r*^2^ > 0.995), and low-density lipoprotein (LDL, A113-1-1, in the range of 0.2-12 mM, *r*^2^ > 0.995) were determined using commercial kits (Nanjing Jiancheng Bioengineering Institute, Nanjing, China).

### 2.7. Western Blot

The PHFs were homogenized in ice-cold RIPA (radio immunoprecipitation) assay buffer (P0013B) with a proteinase inhibitor (ST506, Beyotime Biotechnology, Nanjing, China). Total protein was determined using an Enhanced BCA (bicinchoninic acid) Protein Assay Kit (P0010, Nanjing Jiancheng Bioengineering Institute, Nanjing, China). An equal amount of protein (24 *μ*g) was loaded and separated by 10% SDS-PAGE gel and transferred onto a PVDF membrane (Merck Millipore, Billerica, USA). The membrane was blocked with 5% skimmed milk for 2 h and then incubated overnight at 4°C with corresponding primary antibodies, including mouse anti-GRP78 (1 : 1000, sc-376768), anti-CHOP (1 : 1000, sc-390960), anti-CAMK II (1 : 500, sc-376828), anti-occludin (1 : 200, sc-133256, Santa Cruz Biotechnology Inc., Santa Cruz, USA), anti-ER-*α* (1 : 1000, NB300-560, Novus Biologicals, USA), anti-BAX (1 : 200, EM1203) or GRP75 (1 : 1000, M1603-1), rabbit anti-PPAR gamma (1 : 1000, ET170257), anti-caspase3 (1 : 1000, ET1602-39), anti-Akt1/2/3 (1 : 1000, ET1609-51), anti-phospho-Akt (1 : 1000, ET1607-03), anti-GSK3 beta (1 : 1000, ET1607-71), anti-phospho-GSK3 beta (1 : 1000, ET 1607-60, HuaBio, Hangzhou, China), and mouse anti-VLDLR (1 : 1000, MAB2258, Novus Biologicals, USA), followed by incubation with the horseradish peroxidase-conjugated goat anti-rabbit or anti-mouse secondary antibodies (sc-2004 or sc-2005, Santa Cruz Biotechnology Inc., Santa Cruz, USA) for 1 h at room temperature. *β*-Actin (recognized by mouse anti-*β*-actin, 1 : 1000, R1207-1, HuaBio, Hangzhou, China) was used as the internal control. For protein quantification, images were quantified and analyzed using the ImageJ software; for the grey analysis of proteins, the normalization method was used, and the control group is specified as “1.”

### 2.8. Transmission Electron Microscopy (TEM)

Three types of PHFs (D280-SWFs, D580-SWFs, and ASWFs) were observed with TEM. The follicles were fixed in 2.5% glutaraldehyde at 4°C for 24 h. The PHFs were postfixed with 1% osmium tetroxide (OsO_4_) for 1.5 h at room temperature; then, they were washed in PBS, subsequently dehydrated in ascending concentrations of ethanol, and embedded in LX 112 epoxy resin. Furthermore, the PHFs were sectioned using a Leica EM UC7 Ultramicrotome (Leica Microsystems GmbH Wetzlar, Germany) and mounted on formvar-coated copper grids. The ultrathin sections were stained with 8% aqueous uranyl acetate and alkaline lead citrate for 5 to 10 min, then observed and photographed using a Tecnai G2 Spirit (FEI Company, Hillsboro, USA) with an acceleration voltage of 120 kV at various magnifications. The length of organelles was quantified by the ImageJ software.

### 2.9. RNA Extraction, qRT-PCR, and RNA-Seq Analysis

TRIzol Reagent (Invitrogen, Carlsbad, CA) was used to extract total RNA from follicles. The cDNA was generated from 2 *μ*g total RNA using a HiScript II 1st Strand cDNA Synthesis Kit (Vazyme Biotech Co. Ltd., Nanjing, China), following the manufacturer's protocol. The qRT-PCR was performed using a SYBR® Premix Ex Taq™ kit (Takara, DRR420A, Kyoto, Japan) on an ABI 7500HT Real-time PCR detection system (Applied Biosystems, Foster City, USA), with the following conditions: 95°C for 10 min and then 40 cycles of 95°C for 30 s, 64°C for 34 s, and 72°C for 30 s. Comparisons of expression levels were determined by the 2^−ΔΔCt^ formula method normalized to *β*-actin. The sequences of primers are listed in Table 1. RNA-seq was carried out according to a previous study [38].

### 2.10. Statistical Analysis

All data were presented as the means ± standard error of the means (SEM) and analyzed by one-way analysis of variance (ANOVA) with LSD and Duncan's multiple-range tests using the SPSS 20.0 software. *P* < 0.05 was considered a statistically significant difference.

## 3. Results

### 3.1. Apoptosis in ASWFs

**Figure 1:**
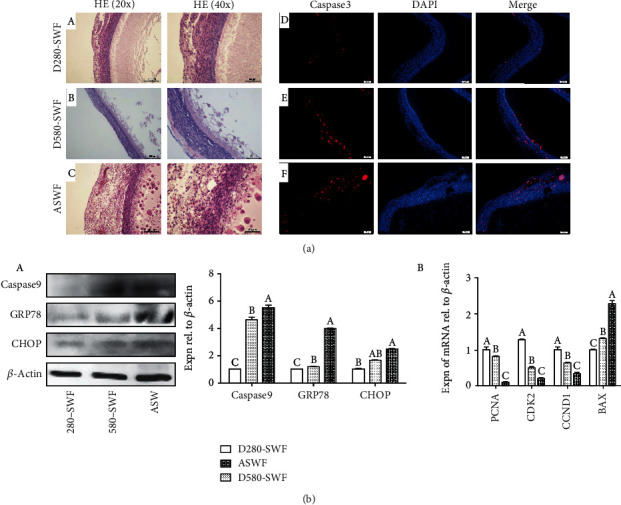
Comparison of SWFs (D280 and D580 hens) and ASWFs (D580 hens). (a) A–C: H&E staining of the SWFs (D280, D580) and ASWFs; D–F: immunofluorescent staining of caspase3 in SWFs (D280, D580) and ASWFs at physiological state. Red: caspase3-labelled cells. Blue: DAPI staining. Scale bar: 50 *μ*m. (b) A: Western blot analysis for the expression of ER-stress-related proteins (caspase9, GRP78, and CHOP) in SWFs (D280 and D580 hens) and ASWFs (D580 hens). B: the expression of *PCNA*, *CDK2*, *CCND1*, and *BAX* mRNAs in SWFs (D280, D580) and ASWFs. Values represent the means ± SEM in each group (*n* = 15). Different lowercase letters indicate significant difference (*P* < 0.05).

Comparing with the healthy SWFs of the same size (2-4 mm), the ASWFs from D580 hens lost their elasticity and became dull and dark with several blooding points (Supplemental Figure 1A). Moreover, at the anaphase of atresia, the follicles shrank, and their shapes collapsed more obviously. From this point, the ASWFs with the same size as healthy follicles were no longer necessarily at the same hierarchy. Therefore, the SWFs at the beginning of atresia were selected for the experiment. H&E staining was used to observe the morphology of PHFs and ASWFs. Results showed that the granulosa layer (GL) of ASWFs was loose and the theca layer (TL) was thickening as compared with SWFs (D280 and D580). However, there was no significant difference in morphology of the SWFs between D580 and D280 hens, since their GLs were all closely arranged, and GCs had regular morphology. In addition, the thickness of the TL was moderate (Figure 1(a) A–C). Moreover, IF images showed that the caspase3 protein was mainly expressed in the GL of PHFs in D580 hens and the TL of ASWFs (Figure 1(a) D–F). Western blot analysis was used to examine the expression of ER-stress-related proteins (caspase9, GRP78, and CHOP). Results showed that those proteins manifested higher expression in ASWFs and SWFs of D580 hens than in those of D280 hens (Figure 1(b) A). Meanwhile, the result of qRT-PCR showed that the expression of *BAX* in ASWFs and SWFs of D580 hens was significantly increased while the expression of the proliferation-associated genes (*PCNA*, *CDK2*, and *CCND1*) decreased significantly as compared with SWFs in D280 hens (Figure 1(b) B).

### 3.2. Comparison of Yolk Depositing Capacity in ASWFs and SWFs

**Figure 2:**
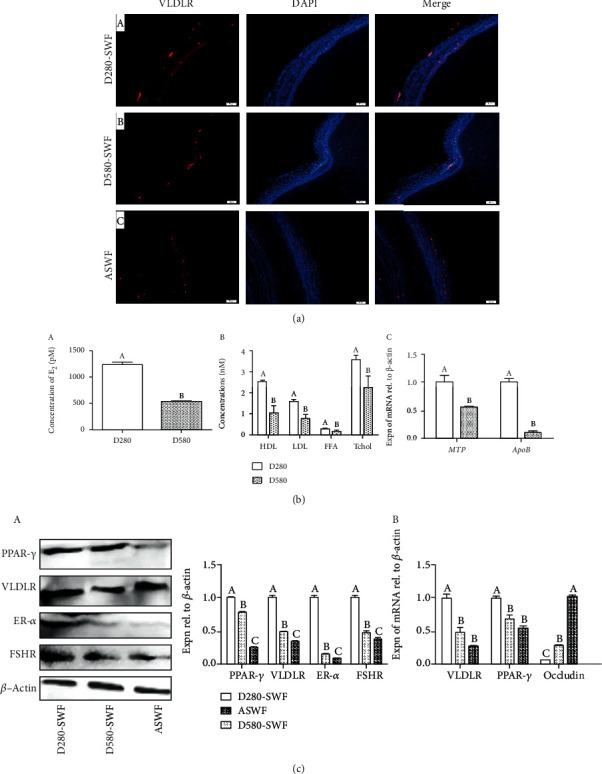
Changes of yolk deposition capacity in SWFs (D280 and D580 hens) and ASWFs (D580 hens). (a) Immunofluorescent staining of VLDLR in SWFs (D280, D580) and ASWFs at physiological state. Red: VLDLR-labelled cells. Blue: DAPI staining. Scale bar: 50 *μ*m. (b) A and B: the levels of serum E_2_ and plasma HDL, LDL, FFA, and Tchol. C: Expression of *MTP* and *ApoB* mRNAs in PHFs of D280 and D580 hens. (c) Analysis of the PPAR-*γ*, VLDLR, ER-*α*, FSHR, and occludin expression in SWFs (D280 and D580 hens) and ASWFs (D580 hens). Values represent the means ± SEM in each group (*n* = 15). Different lowercase letters indicate significant difference (*P* < 0.05).

To determine the changes of the yolk depositing ability of the three kinds of follicles, IF was used to compare the distribution of VLDLR in these follicles. The result showed that the VLDLR protein was mainly expressed in the GL of SWFs of D280 hens, while it was rarely expressed in SWFs of D580 hens and ASWFs (Figure 2(a) A–C). Moreover, the results of the ELISA assay revealed that the levels of serum E_2_ and plasma HDL, LDL, FFA, and Tchol decreased significantly in D580 hens (Figure 2(b) A and B). Meanwhile, the expression level of *MTP* and *ApoB* genes decreased significantly in SWFs of D580 hens (Figure 2(b) C). Furthermore, Western blot analysis showed that PPAR-*γ*, VLDLR, FSH Receptor (FSHR), and ER-*α* proteins exhibited the lowest expression in ASWFs while they displayed the highest expression in SWFs of D280 hens (Figure 2(c) A). Besides, the result of qRT-PCR showed that the expression of *PPAR-γ* and *VLDLR* genes decreased significantly in both ASWFs and SWFs (D580 hens), while the expression of occludin mRNA manifested the highest in ASWF (Figure 2(c) B).

### 3.3. Comparison of Organelle Morphology in GCs

**Figure 3:**
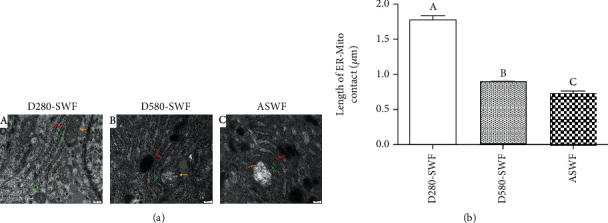
Comparison of organelle morphology in GCs. (a) A–C: the ultrastructure of the SWFs (D280 and D580 hens) and ASWFs (D580 hens). Red arrow: endoplasmic reticulum (ER); yellow arrow: mitochondrion; green arrow: ER-mitochondrion contact points. Scale bar: 200 nm. (b) The length of ER-mitochondrion contact in the SWFs (D280, D580) and ASWFs. Values represent the means ± SEM in each group (*n* = 15). Different lowercase letters indicate significant difference (*P* < 0.05).

Organelle morphology of GCs in three kinds of follicles was observed by TEM. The images displayed that the morphology of the ER in D280-SWFs-GCs had a uniform size and regular density of ribosome in its surface. As for the mitochondrion in D280-SWFs-GCs, it displayed a smooth and flat outer membrane and its inner ridge formed by the inner bulge was visible. However, the ER and mitochondrion in D580-SWFs and ASWFs manifested degenerative signs. The ER displayed a shedding and swollen ribosome, and the mitochondrion lost its regular shape, being swollen and losing its inner ridge. Furthermore, there were more ER-mitochondrion contact points in D280-SWFs than in D580-SWFs and ASWFs (Figure 3(a) A–C). Subsequently, the length of the ER-mitochondrion contact (all touching points in Figure 3) was measured by ImageJ software, and results showed that the point of ER-mitochondrion contact and contact length in groups of D580-SWFs and ASWFs were significantly reduced as compared with those of D280-SWFs (Figure 3(b)).

### 3.4. Establishment of the Follicular Atresia Model

**Figure 4:**
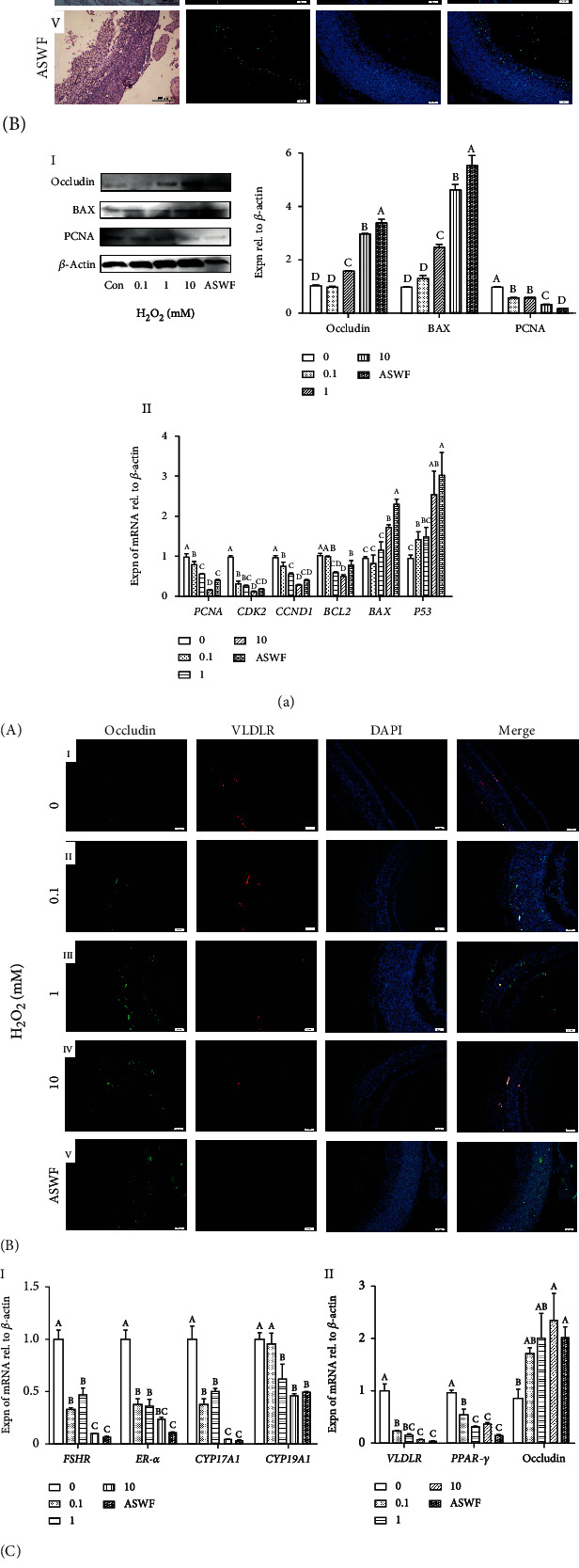
(a) Effect of H_2_O_2_ on cell apoptosis in the atretic follicles. A: H&E staining and TUNEL assay in the SWFs (treated with H_2_O_2_) and ASWFs. Scale bar: 100 *μ*m for H&E staining and 50 *μ*m for TUNEL assay. B-I: Western blot and gray analysis of occludin, BAX, and PCNA expression after H_2_O_2_ treatment. B-II: the expression of *PCNA*, *CDK2*, *CCND1*, *BCL2*, *BAX*, and *P53* mRNAs in the SWFs (treated with H_2_O_2_) and ASWFs. (b) Effect of H_2_O_2_ on yolk deposition capacity and steroidogenesis in the atretic follicle model by H_2_O_2_. A: immunofluorescent labels with occludin (green), VLDLR (red), and DAPI (blue) in the histological sections of the SWFs with different concentrations of H_2_O_2_. Scale bar: 50 *μ*m. B-I: the expression of *FSHR*, *ER-α*, *CYP17A1*, and *CYP19A1* mRNAs. B-II: the expression of yolk deposition-related genes *VLDLR*, *PPAR-γ*, and occludin. C: Western blot and grey analysis of ER-*α* and FSHR after H_2_O_2_ treatment. Values represent the means ± SEM of three replicates in each group. Different lowercase letters indicate significant difference (*P* < 0.05).

It had been previously reported that H_2_O_2_ was capable of inducing apoptosis in the chicken ovary [39]. To examine the impacts of H_2_O_2_ on follicular atresia, SWFs and ASWFs (in vivo) were selected as models. Follicular atresia which was induced by H_2_O_2_ was determined from the following three aspects:

- Cell apoptosis: the results from H&E staining presented that the GL began to lose its complete structure and the arrangement of GCs was no longer dense when the concentration of H_2_O_2_ was higher than 1 mM. Furthermore, the TUNEL assay displayed that the TUNEL-positive cells (green) increased significantly in both TL and GL after the treatment of H_2_O_2_ (Figure 4(a) A, I–V). Meantime, the Western blot analysis displayed that the expression of BAX and occludin proteins gradually increased as the concentration of H_2_O_2_ increased, whereas the expression trend of the PCNA protein showed opposite changes (Figure 4(a) B, I). Moreover, the result of qRT-PCR was consistent with the Western blot analysis (Figure 4(a) B, II)
- Yolk deposition capacity: IF showed that occludin and VLDLR proteins were mainly expressed in the GL, and the occludin expression was upregulated with the increasing stepwise concentration of H_2_O_2_, while the expression of VLDLR showed a contrary trend (Figure 4(b) A, I–V). The result of qRT-PCR revealed that the expression of genes in SWFs was comparable with ASWFs after the treatment of 10 mM H_2_O_2_ (Figure 4(b) B, I). However, the expression of *PPAR-γ*, *VLDLR*, and occludin mRNAs in SWFs was similar with the ASWFs when the concentration of H_2_O_2_ was above 1 mM (Figure 4(b) B, II)
- Hormone synthesis: the expression of hormone receptor proteins (ER-*α* and FSHR) was determined by Western blot analysis. Results showed that the expression of ER-*α* and FSHR in SWFs was comparable with ASWFs when the concentration of H_2_O_2_ is higher than 1 mM (Figure 4(b) C). Based on these results, 1 mM H_2_O_2_ was selected for the establishment of the ASWF model.

### 3.5. Effect of Met on Inhibiting Apoptosis in Follicular Atresia

**Figure 5:**
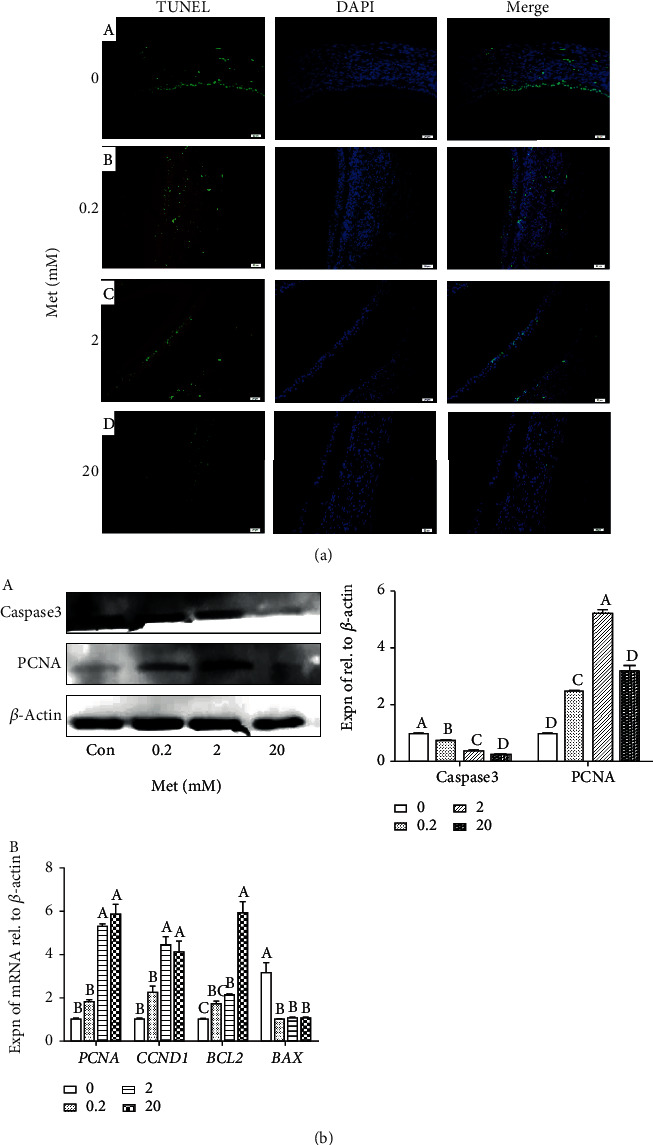
Effect of Met on cell proliferation and apoptosis of ASWFs (H_2_O_2_-induced). (a) TUNEL assay in ASWFs after treatment with different concentrations of Met. Scale bar: 50 *μ*m. (b) Effect of different concentrations of Met on expression of proliferation and apoptosis-related genes and proteins in ASWFs. Values represent the means ± SEM of three replicates in each group. Different lowercase letters indicate significant difference (*P* < 0.05).

To investigate the relieving effect of Met on H_2_O_2_-induced follicular atresia, the dose of Met was screened from four concentrations (0, 0.2, 2, and 20 mM). The result of the TUNEL assay revealed that the treatment of 0.2 and 2 mM Met inhibited the apoptosis of GCs in ASWFs (H_2_O_2_ induced), by comparing with the control group (Figure 5(a) A–D). As for protein aspects, the 2 mM Met significantly increased the expression of PCNA, while 2 mM Met represented the finest effect on inhibiting caspase3 (Figure 5(b) A). Furthermore, treatments of 2 and 20 mM Met upregulated the expression of *PCNA* and *CCND1* and downregulated the expression of *BAX* in the mRNA level (Figure 5(b) B). In conclusion, the concentration of 2 mM Met was selected as the optimal dose.

### 3.6. Differentially Expressed Gene (DEG) Profiles between D280 and D580 SWFs

**Figure 6:**
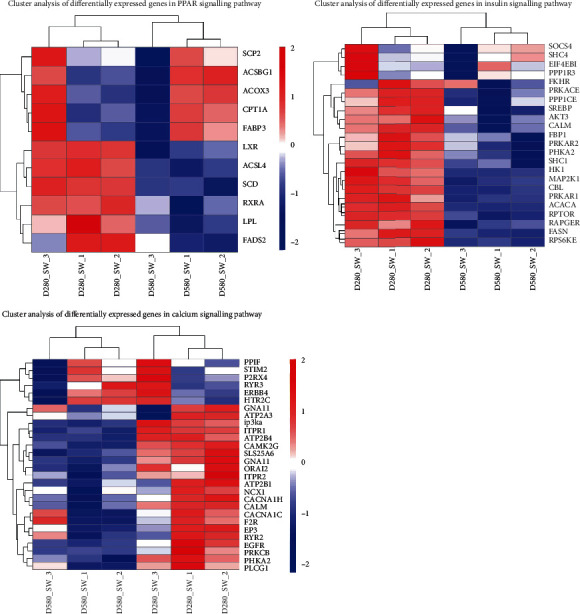
Heatmap of DEGs in calcium, PPAR, and insulin pathways.

Cluster analysis of DEGs in the calcium, PPAR, and PI3K/AKT pathways indicated that the gene expression patterns of D280 SWFs were different from D580 (Figure 6). Many genes in these three pathways displayed significantly different expression between D280 and D580 SWFs. For example, the expression of *PIK3CD*, *AKT3*, *NCX1*, *EGFR*, *SCP2*, *CPT1A*, and *FABP3* mRNAs in D580 SWFs decreased significantly as compared with D280 SWFs. However, *ATP2B2*, *PTK2B*, *ITPR3*, and *NCX1* mRNAs in D580 SWFs increased significantly as compared with D280 SWFs (Table 2).

### 3.7. Mechanisms of Met in Antiaging/Atresia

**Figure 7:**
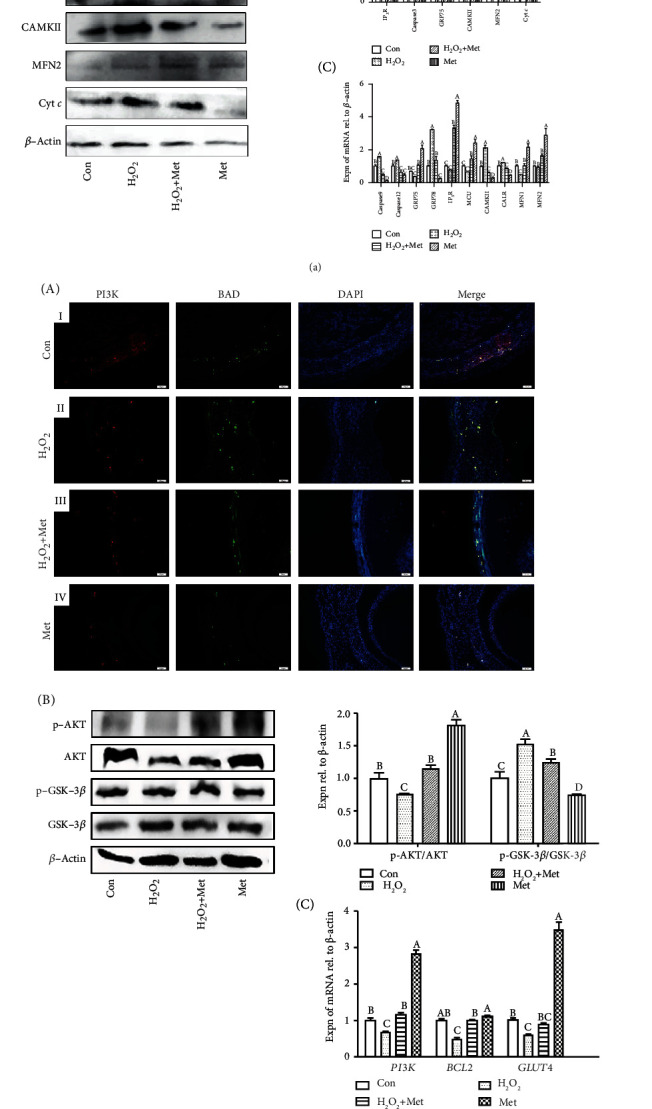
(a) Mechanism of antiaging/atresia of Met via the calcium ion (Ca^2+^) transport signaling pathway in the H_2_O_2_-induced ASWFs. A: the effect of Met on the expression of GRP75, Cyt *c*, and MFN2 with IF and IHC analyses. Scale bar: 50 *μ*m. B: Western blot and grey analysis of IP_3_R, caspase3, GRP75, CAMKII, MFN2, and Cyt *c* expression in ASWFs. C: effect of Met on expression of Ca^2+^ transport-related genes. (b) Mechanism of antiaging/atresia of Met via the insulin signaling pathway. A: the effect of Met on expression of PI3K (red), BAD (green), and DAPI (blue) with IF. Scale bar: 50 *μ*m. B: Western blot and grey analysis of p-AKT/AKT and p-GSK-3*β*/GSK-3*β* expression in ASWFs. C: effect of Met on expression of *PI3K*, *BCL2*, and *GLUT4* mRNAs in ASWFs. (c) Mechanism of antiaging/atresia of Met via the lipid metabolism signaling pathway. A: Western blot and grey analysis of PPAR-*γ*, occludin, and VLDLR expression in ASWFs. B: qRT-PCR analysis of *PPAR-γ*, occludin, and *VLDLR* expression in ASWFs. Values represent the means ± SEM of three replicates in each group. Different lowercase letters indicate significant difference (*P* < 0.05).

From IF staining, GRP75 and Cyt *c* proteins were mainly located in GL, and a small amount was distributed in TL of follicles. The expression of GRP75 decreased, while the expression of Cyt *c* increased after H_2_O_2_ treatment. However, the expression of GRP75 and Cyt *c* was similar to the control group after the Met treatment (Figure 7(a) A, I–IV). The result of IHC of mitochondrion fusion protein 2 (MFN2) showed similar results with GRP75 in IF (Figure 7(a) A, V–VIII). Western blot and qRT-PCR were used to determine the function of the calcium ion transport signaling pathway in this study. After the Met treatment, these proteins' (IP_3_R, caspase3, CAMKII, and Cyt *c*) expression decreased significantly while the expression of MFN2 and GRP75 showed an opposite trend (Figure 7(a) B). Results of qRT-PCR showed that the expression trend of caspase9, *GRP75*, *CAMKII*, and *MFN2* mRNAs was similar with the Western blot detection (Figure 7(a) C). Meanwhile, the insulin signaling (PI3K/AKT) pathway was suppressed after the H_2_O_2_ treatment. Furthermore, the expression of the BAD protein in TL and GL decreased after the Met treatment, while the expression of PI3K experienced a significant increase (Figure 7(b) A). Results of Western blot displayed that Met decreased the ratio of p-GSK-3*β*/GSK-3*β* and improved the ratio of p-AKT/AKT (Figure 7(b) B). Moreover, qRT-PCR revealed that the expression patterns of *PI3K*, *BCL2*, and *GLUT4* mRNAs were similar to PI3K in IF (Figure 7(b) C). Likewise, the results of Western blot and qRT-PCR showed that Met restored the decrease of VLDLR and PPAR-*γ* that was induced by H_2_O_2_, while occludin displayed an opposite expression trend (Figure 7(c) A and B).

### 3.8. Promoting Effect of Met on Cell Proliferation Involved in Activating PI3K in GCs of ASWFs

**Figure 8:**
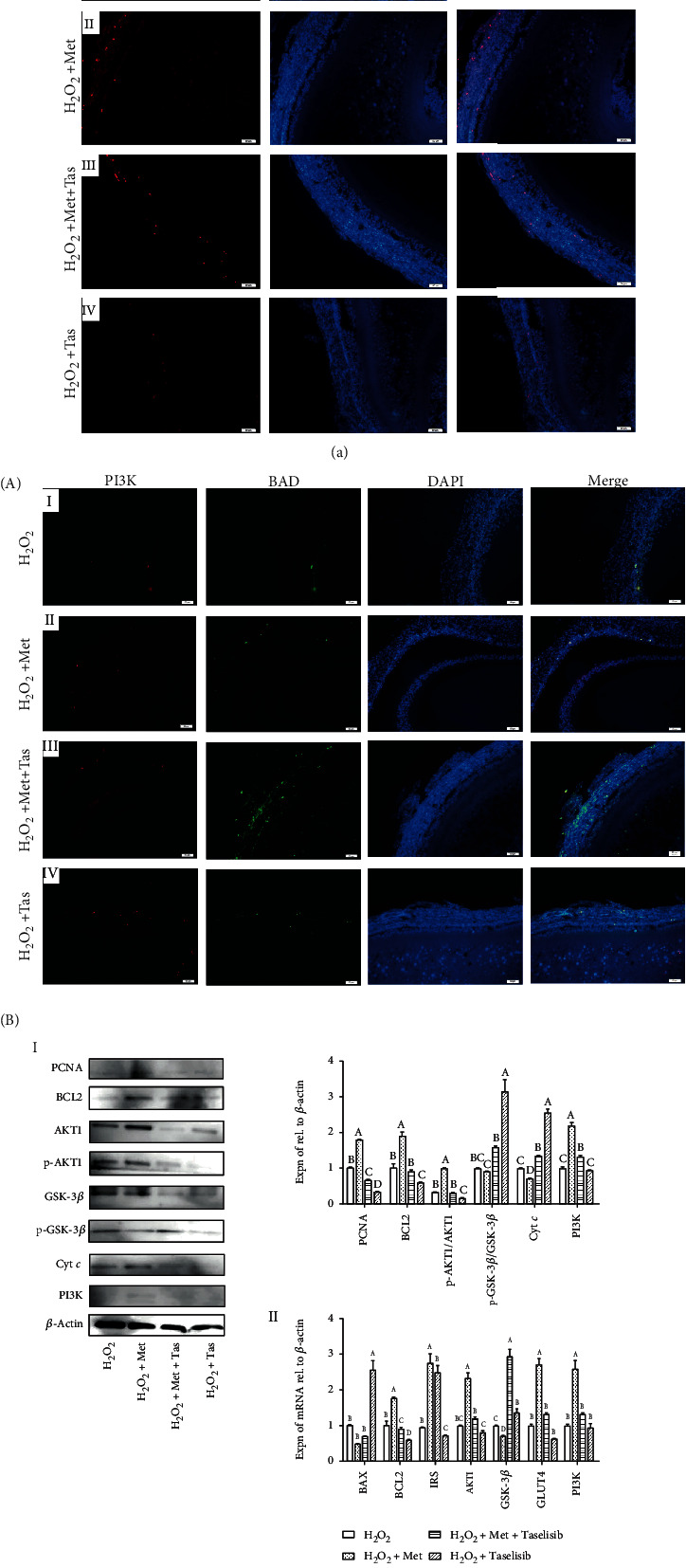
(a) Effect of Met and Taselisib (Tas) on BrdU incorporation in proliferating cells in the H_2_O_2_-induced ASWFs. A: red—BrdU-labelled cells; blue—DAPI staining. Scale bar: 50 *μ*m. (b) Function of PI3K in Met is to stimulate cell proliferation in GCs of ASWFs. A: effect of Met and Tas on expression of PI3K (red) and BAD (green) in ASWFs. Blue: DAPI staining. Scale bar: 50 *μ*m. B-I: Western blot and grey analysis of PCNA, BCL2, p-AKT1, AKT1, p-GSK-3*β*, GSK-3*β*, Cyt *c*, and PI3K expression in ASWFs. B-II: qRT-PCR analysis of the *BAX*, *BCL2*, *IRS*, *AKT1*, *GSK-3β*, *GLUT4*, and *PI3K* mRNA expression in ASWFs. Values represent the means ± SEM of three replicates in each group. Different lowercase letters indicate significant difference (*P* < 0.05).

To investigate the function of Met in the PI3K/AKT signaling pathway, Taselisib (a kind of PI3K inhibitor) was used in the culture of ASWFs [40]. Results of BrdU showed that the BrdU-positive cells (red) in GL decreased significantly after the 0.3 mM Taselisib treatment (Figure 8(a) A). Meantime, IF results demonstrated that Taselisib suppressed the PI3K expression and increased the number of BAD-positive cells in both TL and GL. Nevertheless, Met was capable of alleviating these phenomena (Figure 8(b) A, I–IV). Moreover, Western blot analysis displayed that Taselisib upregulated the expression of Cyt *c* and the ratio of p-GSK-3*β*/GSK-3*β*, and it downregulated the expression of BCL2, PCNA, and PI3K as well as the ratio of p-AKT/AKT. However, Met treatment reversed these changes significantly (Figure 8(b) B, I). Besides, the result of qRT-PCR further confirmed the above results (Figure 8(b) B, II).

### 3.9. Stimulating Effect of Met on E_2_ Secretion in D580 Hens

**Figure 9:**
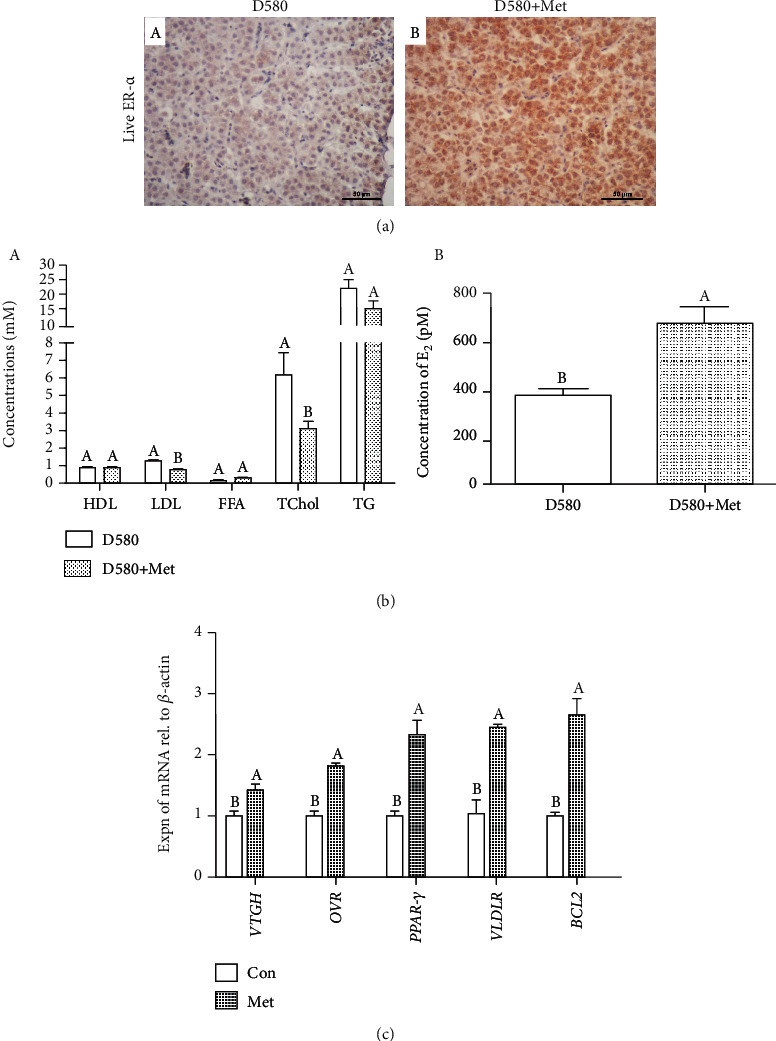
Met promoted yolk synthesis by stimulating estrogen secretion in D580 hens. (a) The number of ER-*α*-positive cells (brown) in the liver of D580 hens was increased by Met. Scale bar: 50 *μ*m. (b) Effect of Met on levels of plasma HDL, LDL, FFA, Tchol, TG, and serum E_2_ in D580 hens. (c) Effect of Met on expression of yolk deposition-related genes (*VTGII*, *OVR*, *PPAR-γ*, and *VLDLR*) and *BCL2* in D580 hens. Values represent the means ± SEM in each group (*n* = 15). Different lowercase letters indicate significant difference (*P* < 0.05).

To examine the effect of Met in vivo, the Met sustained-release tablets were orally administered for 7 days (based on the rate of egg reproduction, Supplemental Figure 1B). The result of IHC showed that Met increased the ER-*α*-positive cells (brown) in the liver of D580 hens (Figure 9(a) A and B). However, no significant changes of plasma HDL, FFA, and TG levels were observed, and LDL and Tchol showed a significant decline after the Met treatment (Figure 9(b) A), while serum E_2_ levels were significantly higher in the Met group as compared with the control group (Figure 9(b) B). Meanwhile, the expression of yolk deposition-related genes (*VTGII*, *OVR*, *PPAR-γ*, and *VLDLR*) as well as *BCL2* in follicles displayed a significant increase after Met treatment (Figure 9(c)).

## 4. Discussion

The greatest feature of the ovarian degeneration in mammals is the remarkable increase in the number of AFs [41]. This phenomenon is similar in poultry [42]. Follicular atresia may occur in any stage of follicular development and is mainly caused by apoptosis of germ cells or somatic cells [6]. In the poultry industry, an increase in the number of AFs directly reduces egg production [4]. Decreasing the number of AFs is bound to increase the chances of follicles developing into hierarchical follicles, thereby raising egg production [7]. The expressions of genes or proteins related to yolk deposition and apoptosis among D280-SWFs, D580-SWFs, and ASWFs (D580) are compared, and results show that the capacity of yolk deposition declines in ASWFs, which is consistent with a previous study [43].

During the period of follicular degeneration, there were other elements that played a crucial role in it. FSH can induce differentiation of follicular GCs and increase the formation of the luteotropic hormone receptor, and then stimulate the release of steroids from GCs, ultimately restricting apoptosis and follicular atresia [44]. In addition to FSH, E_2_ also plays an important role in inhibiting follicular atresia. This study showed that the expression of FSHR and ER-*α* decreased in ASWFs, which is similar to previous reports [12, 45]. Based on previous studies [3, 7, 46], the follicle atretic model was evaluated from three aspects (yolk deposition capacity, apoptosis, and hormone receptor production). After treatment with 1 mM H_2_O_2_, the expression of FSHR, ER-*α*, PPAR-*γ*, VLDLR, and occludin in SWFs was similar to that in ASWFs. These results demonstrate that the model was successfully established.

As for the GC apoptosis, the expression of GRP78 (one of the markers of ER-stress) would increase when GCs of D580 hens undergo apoptosis [7]. Results of the current study showed that the expression of GRP78, CHOP, and caspase9 was increased in ASWFs. At the same time, the TEM images demonstrated that the rough ER and mitochondrion in D580-ASWF-GCs manifested degeneration. Those results suggested that ER-stress and mitochondrion apoptotic pathways participated in follicular atresia. Meanwhile, the calcium ion imbalance of ER could be seen as the main reason of inducing ER-stress [13]. Therefore, it was important to regulate Ca^2+^ concentration in the ER. MAM is the functional domain in both organelles (ER and mitochondrion) that is involved in Ca^2+^ exchange [14]. GRP75 is an important part of MAM, and it has been reported that knockdown of GRP75 reduced the ability of the ER to carry Ca^2+^ to the mitochondrion [19]. Moreover, MFN2 that participated in the formation of the integral functional unit of MAM decreased the release of Cyt *c* and inhibited the mitochondrion apoptosis pathway [47, 48]. At the same time, the BAX homodimer of the Bcl2 family existed on the mitochondrial surface, and the unphosphorylated BAD (ATK downstream transcription factor) induced the nonaggregation of BAX and further decreased mitochondrion membrane potential by increasing mitochondrion calcium level (calcium overload). These changes finally led to the release of Cyt *c* and ultimately activated caspase and induced apoptosis of mitochondrion [20, 21]. It was the junction of the calcium signaling pathway with PI3K/AKT.

Met can be seen to have a positive contribution to antiaging. For instance, it decreased follicle loss in aged mice and reduced the oxidative stress of the ovary [31, 32]. Besides, Met was demonstrated to significantly reduce the cellular apoptosis in AFs [49]. After the Met treatment, the expressions of IP_3_R, caspase3, and Cyt *c* were significantly decreased, while the expressions of MFN2 and GRP75 were increased. It indicated that Met could inhibit the mitochondrion apoptosis pathway by decreasing the expression of Cyt *c* and suppressing caspase3 which played a vital role in GC apoptosis [13]. This function mainly benefited from balancing the Ca^2+^ concentration between the ER and the mitochondrion. The survival of GCs provided the developmental motivation of PHFs, since they secreted vital supporting elements [7, 50–52]. However, it was still unclear whether Met could inhibit apoptosis by rebuilding MAM function; a deeper exploration of the 3D morphology of ER and mitochondrion may be needed. In another aspect, our study found that the expression of PI3K and the p-AKT/AKT was decreased significantly, while p-GSK-3*β*/GSK-3*β* was increased in the model of follicular atresia that was induced by H_2_O_2_. In this model, the antioxidant capacity was decreased in ASWFs [53, 54]. This result was consistent with H_2_O_2_-induced oxidative stress [55]. Moreover, the number of PI3K-positive cells in GL was increased in the Met-treated group with restriction of BAD expression. A consistent result reported that the inactivation of PI3K/AKT induced GC apoptosis [8]. Furthermore, after the treatment of Taselisib (a PI3K inhibitor), Met could also activate PI3K to some extent, thereby maintaining the activity of the PI3K/AKT signaling pathway.

**Figure 10:**
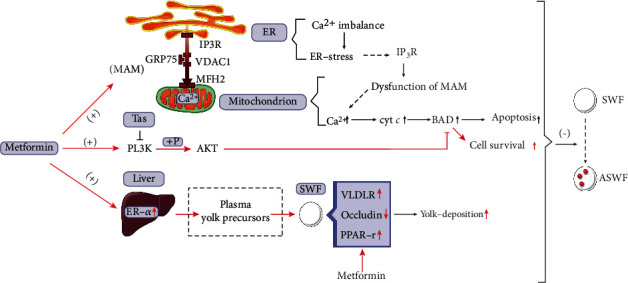
Mechanism of Met preventing follicular atresia in aging chickens.

Met also played an essential part in regulating the yolk formation/deposition. After one week of continuous oral Met administration, the level of serum E_2_ and the liver ER-*α* increased significantly, similar to previous reports [56]. However, the egg-yolk precursors in plasma (HDL, FFA, and TG) had a significant change except for LDL and Tchol. The in vitro experiment showed that Met stimulated the expression of VLDLR and PPAR-*γ*, while it inhibited the expression of occludin and enhanced the capacity for yolk deposition. Furthermore, it had been reported that VLDLR which existed on the surface of migrating neuroblasts could activate PI3K/AKT through p-Dab1 [25]. In this study, we found that the expression trend of VLDLR and PI3K/AKT is similar, and we supposed that Met may activate PI3K through regulating the expression of VLDLR. Moreover, Met was demonstrated to stimulate lipid metabolism and reduce the level of blood triglyceride [57, 58]. By combining the results of this experiment, Met was speculated to enhance the capacity of yolk formation and accelerate egg-yolk precursor transit from the plasma to the ovarian follicles. Whether this acceleration is involved in fastened yolk deposition and lipid metabolism needs further exploration. Based on those arguments, Met performs an effective action in inhibiting follicular atresia by regulating the cooperation of multiple signaling pathways (Figure 10).

## 5. Conclusion

In this study, a follicular atresia model was established to explore the mechanism of Met in inhibiting follicular atresia in chickens. Met was able to inhibit the expression of BAD and decrease apoptosis by activating PI3K. Meanwhile, Met may intervene in the ER-mitochondrion connection to attenuate ER-stress and mitochondrion apoptosis pathways to prevent apoptosis and follicular atresia. Met also promoted yolk formation in the liver and yolk deposition in the ovarian follicles. In summary, Met was capable of relieving ovarian aging and follicular atresia through the coordination of multiple signaling pathways. This study may provide a new measure for extending the laying period of poultry by dietary Met supplementation.

## Figures and Tables

**Table 1:** The primers of qRT-PCR.

Gene name	Accession number	Primer sequence (5′-3′)	Product length (bp)
*PCNA*	NM_204170.2	F: GGGCGTCAACCTAAACAGCA R: AGCCAACGTATCCGCATTGT	97
*CCND1*	NM_205381.1	F: CCTCAAGAAAAGCCGGTTGC R: CTGCGGTCAGAGGAATCGTT	86
*CDK2*	NM_001199857.1	F: TCCGTATCTTCCGCACGTTG R: GCTTGTTGGGATCGTAGTGC	183
*MTP*	NM_001109784.2	F: GCAGATGGACAGAGTTGGCT R: TTCCCTCTCCTCGCAGTGTA	93
*ApoB*	NM_001044633.1	F: AGGTAGAGGCAGGACGCATA R: AGAATGCTACGTCCCACACG	268
*VLDLR*	NM_205229.1	F: ATGGCCAGGATCGTAGACTT R: TCATTTATCTGAGGAGCAGG	292
*PPAR-γ*	NM_001001460.1	F: CAAGGCAGCGGCAAAATAAC R: GTGCCCATAAATGATGGCCTAA	187
Occludin	NM_205128.1	F: CTCTGGGAAGGGCTGAGGT R: GCCTTCCCAAAAAGCCCTGA	170
*BCL2*	NM_205339.2	F: ATCGTCGCCTTCTTCGAGTT R: ATCCCATCCTCCGTTGTCCT	150
*P53*	NM_205264.1	F: AACCATTGCTGGAACCCACT R: GCCAGTTGCTGTGATCCTCA	99
*ER-α*	NM_205183.2	F: TAGTTCCGCTCTACGACCTCTT R: AGTTGGTTTCGGTTCTCCTCTT	106
*CYP17A1*	NM_001001901.2	F: GGAGCTGACAGATGACCACC R: TCTTCTGGACCTCGGGGRAG	125
*CYP19A1*	NM_001001761	F: CCTCTGCTGGAGATGGTTTT R: GCTGATCCACTTTAGTCACTCTGA	68
*IP3R*	KY290442.1	F: CTACTTGAAGGCGGAAACCCT R: GCCCGCCATGTCTGAAGTAT	162
*MCU*	XM_025151715.1	F: CTGAAATGCATGGCTGCACG R: GTTTGAGGGTGAACTGGCAAC	164
*CAMKII*	XM_015288323.2	F: AGATGAGGATCTGAAAGTGCGT R: TTCCCCAGTGCTTCAGGTTC	144
*MFN1*	NM_024200.4	F: CGGAGTGAGTGTCCGCTG R: GTGCTTCAGTGGAGATACCGT	180
*MFN2*	XM_015297206.2	F: GTGGTTGTGTTTTGTTACTTCCG R: AGGGACATTGCGTTTTCAGG	275
*PI3K*	NM_001004410.1	F: ACACGTTCTTGTGCTGGCTA R: TAAGACAAAGGGCACACGCT	177
*GLUT4*	XM_025145523.1	F: CGTGGGTGAGCTTTCCAGAT R: CATGTAGCGCCCAAAGAACG	86
*IRS*	XM_003641084.4	F: GTCATCCCGCTCTACCAGTG R: TTCTCCGCCAGCATAGCAAA	110
*AKT1*	NM_205055.1	F: GCGAGAAGGGGAGGAGGA R: CCTGAACTCCAGCAGAAGGG	272
*GSK-3β*	XM_004938179.3	F: ATGGACTAAGGTCTTCCGGC R: CTCTCGCCCGTTTGGTAACT	166
*VTGII*	NM_001031276	F: TTGCAAGCTGATGAACACACAC R: GATTGCTTCATCTGCCAGGTC	192
*OVR*	X95100.1	F: TGAAGCCTGCTGTGACTGTG R: AAGTGACTGACAGGAGTGAGC	249
*GRP78*	NM_205491.1	F: GAATCGGCTAACACCAGAGGA R: CGCATAGCTCTCCAGCTCATT	118
*GRP75*	NM_001006147.1	F: GCAGCAGGCTTCCTTGAAAC R: ATTCCGCCCATTTCTGCTCA	198
*FSHR*	NM_205079.1	F: ACCTGCCTGGATGAGCTAAA R: ATCCATGACTTGGCAGGAAG	136
*CALR*	XM_025145796.1	F: GTGGAGACCCCGACAGATTG R: GTGGAGACCCCGACAGATTG	99
Caspase9	XM_424580.6	F: GCTTGTCCATCCCAGTCCAA R: TGCTGCTGACACCTTCACCATTC	95
*β*-Actin	NM_205518	F: ACACCCACACCCCTGTGATGAA R: TGCTGCTGACACCTTCACCATTC	136

**Table:** (a) Insulin signaling pathway

Gene ID	Gene symbol	Log_2_FC	*P* adj	Description
Upregulated genes				
ENSGALG00000007919	EIF4E2	0.20281	0.005635	Translation initiation factor eIF-4e
ENSGALG00000006418	PDPK1	0.21168	0.00028551	Tyrosine-protein kinase, catalytic domain
ENSGALG00000001267	MAP2K2	0.18059	0.020795	Serine-threonine/tyrosine-protein kinase catalytic domain
ENSGALG00000002485	PHKG1	0.21809	0.0065997	Phosphorylase kinase, gamma catalytic subunit
ENSGALG00000007373	PRKAB1	0.20238	0.0012886	Immunoglobulin E-set
ENSGALG00000026692	N-RAS	0.1801	0.0062565	Small GTP-binding protein domain
ENSGALG00000010440	MKNK1	0.3733	0.002099	Tyrosine-protein kinase, catalytic domain
ENSGALG00000001678	PRKAB2	0.26435	0.01324	Association with the SNF1 complex (ASC) domain
ENSGALG00000006885	H-RAS	0.32313	2.3544*E* − 10	Small GTPase superfamily, Rab type
ENSGALG00000027275	PPP1R3B	0.31903	4.6517*E* − 08	Putative phosphatase regulatory subunit
Downregulated genes				
ENSGALG00000002583	PIK3CD	-0.41721	0.0039568	Phosphoinositide 3-kinase, accessory (PIK) domain
ENSGALG00000007023	PRKAR2A	-0.3031	0.0003467	cAMP-dependent protein kinase regulatory subunit
ENSGALG00000010709	AKT3	-0.28405	0.049919	Pleckstrin homology domain
ENSGALG00000027188	SREBP-1	-0.32066	0.026855	Tumor necrosis factor-like domain
ENSGALG00000005198	RPS6KB1	-0.31356	0.0034049	Tyrosine-protein kinase, catalytic domain

**Table:** (b) Calcium signaling pathway

Gene ID	Gene symbol	Log_2_FC	*P* adj	Description
Upregulated genes				
ENSGALG00000003901	P2RX4	0.42582	2.2908*E* − 08	P2X2 purinoceptor
ENSGALG00000004096	CHRNA7	0.80665	0.000017539	Nicotinic acetylcholine receptor
ENSGALG00000009808	VT4	1.1702	0.015681	Vasopressin receptor
ENSGALG00000002485	PHKG1	0.21809	0.0065997	Phosphorylase kinase, gamma catalytic subunit
ENSGALG00000007779	ADCY9	0.14706	0.024756	Nucleotide cyclase
Downregulated genes				
ENSGALG00000004829	ATP2B2	-0.32696	0.0035202	Cation-transporting P-type ATPase, C-terminal
ENSGALG00000004792	PLCB2	-0.55479	0.0041353	Phospholipase C, phosphatidylinositol-specific, EF-hand-like
ENSGALG00000016564	PTK2B	-1.2119	0.043151	FERM central domain
ENSGALG00000003750	PLCG1	-0.35419	0.0010363	Phospholipase C, phosphatidylinositol-specific, Y domain
ENSGALG00000003149	ITPR3	-0.93013	0.00049355	Inositol 1,4,5-trisphosphate-binding protein receptor
ENSGALG00000010013	EDNRA	-0.71035	1.0324*E* − 08	Endothelin receptor A
ENSGALG00000001564	ATP2A3	-0.73625	0.0012319	P-type ATPase
ENSGALG00000008544	NCX1	-1.0991	0.000055847	Sodium/calcium exchanger, isoform 1
ENSGALG00000012363	EGFR	-0.18932	0.012951	Insulin-like growth factor binding protein, N-terminal
ENSGALG00000014071	ITPR2	-0.51033	7.7531*E* − 06	Armadillo-type fold
ENSGALG00000013929	PDGFRA	-0.52088	0.0036094	Tyrosine-protein kinase, catalytic domain
ENSGALG00000016691	SLC25A6	-0.14184	0.016577	Adenine nucleotide translocator 1
ENSGALG00000005805	PLCD1	-0.49646	0.005357	Pleckstrin homology domain
ENSGALG00000009095	LHCGR	-0.44467	5.2239*E* − 09	G protein-coupled receptor, rhodopsin-like
ENSGALG00000021313	PDGFRB	-0.43421	0.0035472	Protein kinase-like domain
ENSGALG00000006014	PRKCB	-0.57054	0.000064666	Protein kinase, C-terminal
ENSGALG00000008875	PLCB1	-0.44568	0.0055755	Phospholipase C, phosphatidylinositol-specific, Y domain

**Table:** (c) Yolk deposition signaling pathway

Gene ID	Gene symbol	Log_2_FC	*P* adj	Description
Upregulated				
ENSGALG00000007077	CPT1A	0.28028	7.9849*E* − 06	Acyltransferase ChoActase
ENSGALG00000010652	SCP2	0.12657	0.030231	Thiolase-like
ENSGALG00000003286	ACSBG1	0.87572	0.030231	AMP-dependent synthetase
ENSGALG00000000620	FABP3	0.52972	7.9382*E* − 14	Cytosolic fatty-acid binding
ENSGALG00000015591	ACOX3	0.33528	0.0039558	Acyl-CoA dehydrogenase
Downregulated				
ENSGALG00000015425	CPT1A	-0.25383	0.00071396	Triacylglycerol lipase family
ENSGALG00000007178	SCP2	-0.47547	1.563*E* − 15	Fatty acid
ENSGALG00000008088	ACSBG1	-0.21285	0.0004066	AMP-dependent synthetase
ENSGALG00000005739	FABP3	-0.19134	0.0028215	Fatty acid desaturase, type 1, core
ENSGALG00000002626	ACOX3	-0.31722	0.0075549	Nuclear hormone receptor
ENSGALG00000008202	CPT1A	-0.15085	0.0034285	Ligand binding

## Data Availability

No data were used to support this study.

## Supplementary Materials

Supplementary Materials Supplemental Figure 1: morphological comparison between ASWF and SWF (A) and changes in the laying rate after Met administration (B).
