# Supplementary material for: Metformin Prevents Follicular Atresia in Aging Laying Chickens through Activation of PI3K/AKT and Calcium Signaling Pathways
Source: Oxid Med Cell Longev. 2020 Nov 26;2020:3648040. doi: 10.1155/2020/3648040 (PMC7718058; doi:10.1155/2020/3648040)
Supplement: Supplementary Materials — Supplemental Figure 1: morphological comparison between ASWF and SWF (A) and changes in the laying rate after Met administration (B). [file 3648040.f1.docx]

**A** ASWF SWF


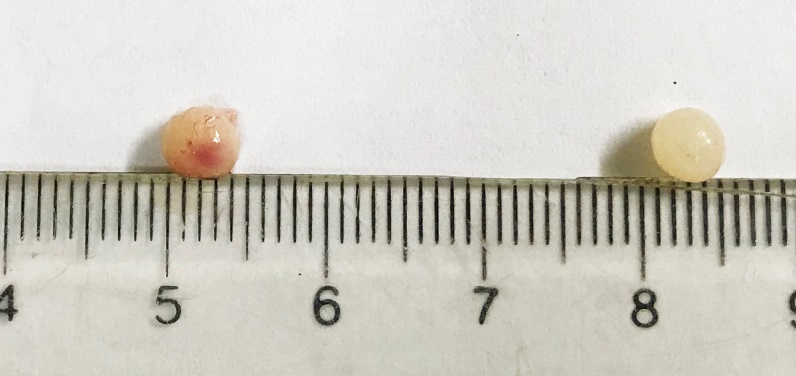


Follicular morphology

**B**


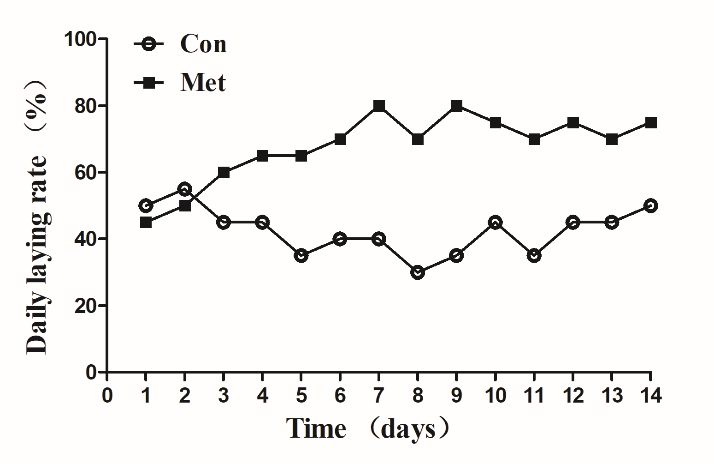


Supplemental Figure 1 : Morphological comparison between ASWF and SWF (A) and changes in the laying rate after Met administration (B).
